# Editorial: Gene regulation mediated by competing RNA: From benchside to bedside

**DOI:** 10.3389/fgene.2022.1085155

**Published:** 2022-11-23

**Authors:** Yumei Luo, Detu Zhu, Jian-Hong Fang

**Affiliations:** ^1^ Department of Obstetrics and Gynecology, Key Laboratory for Major Obstetric Diseases of Guangdong Province, The Third Affiliated Hospital of Guangzhou Medical University, Guangzhou, China; ^2^ Guangzhou Laboratory, Guangzhou, China; ^3^ Institute of Biological Products, National Institutes for Food and Drug Control, Beijing, China; ^4^ Department of Genetic Medicine, Weill Cornell Medical College, New York, NY, United States; ^5^ MOE Key Laboratory of Gene Function and Regulation, School of Life Sciences, Sun Yat-sen University, Guangzhou, China

**Keywords:** microRNA, long non-coding RNA, circular RNA, competing endogenous RNA network, gene regulation, CRISPR, SARS-CoV-2

In addition to DNA methylation and chromatin structure, another important level of epigenetic regulation for gene expression is non-coding RNA (ncRNA) (Luo et al., 2019). NcRNAs facilitate post-transcriptional gene regulation *via* competitively interacting with the regulatory elements of target genes (Zhang et al., 2022). The course of various biological processes, the development of diseases, and the susceptibility to therapy are all significantly influenced by competitive ncRNA gene regulation (Panni et al., 2020). Numerous studies have demonstrated that important functional genes could be regulated by these ncRNAs *via* competing endogenous RNA (ceRNA) network (Salmena et al., 2011; Tay et al., 2011; Tay et al., 2014). Moreover, a large number of therapeutic means have been developed based on these mechanisms (Luo and Zhu, 2014). Thus, competitive RNA is expected to be used not only for basic research but also as a promising tool for applications in therapy. In this Research Topic, the essential functions and mechanisms of ncRNAs are further identified and investigated.

Circular RNAs (circRNAs) are ncRNAs that play a regulatory role in many biological processes, such as cell proliferation, senescence, and apoptosis (Visci et al., 2020). Many studies have found that circRNAs can play their regulatory role as microRNA (miRNA) sponges in human physiological and pathological processes (Peng et al., 2021). Whether as a diagnostic biomarker or as a therapeutic target, circRNAs have important values for studying the pathogenesis of diseases owing to their unique properties.

Aberrant alterations in nucleus pulposus cells (NPCs) are the most significant aspect of the pathological process of intervertebral disk degeneration (IDD) (Fontana et al., 2015; Oichi et al., 2020). In this context, Li et al. investigated circRNAs and miRNAs associated with NPC metabolism under the IDD condition. They identified that circ_0040039 might aggravate IDD by stabilizing miR-874-3p and thus upregulate the miR-874-3p-ESR1 pathway, which lead to NPC degeneration and worsen IDD. In another study, Duan et al. demonstrated that circMTO1 could accelerate the progression of polycystic ovary syndrome (PCOS) by increasing MCL1 expression through interaction with miR-320b, offering novel insights into the development of diagnosis and treatment for PCOS. Furthermore, Cai et al. revealed that hsa_circRNA_0001400 is substantially expressed in cervical cancer tissues and promotes tumor progression *via* the circRNA_0001400-miR-326-Akt pathway. Knockdown of circRNA_0001400 by siRNA might potentially developed as a new method for cervical cancer therapy.

Long non-coding RNAs (lncRNAs), like circRNAs, can serve as miRNA sponges to provide indirect regulation of gene expression (Zhang et al., 2018). Besides, artificial lncRNAs have recently drawn a lot of interest as novel disease therapy tools to be utilized in gene regulatory activities (Tang et al., 2016).

Atrial fibrillation (AF), the most prevalent kind of arrhythmia, poses a great challenge in patient diagnosis and therapy (Hindricks et al., 2021), and further research into the pathophysiology of AF is of urgent need. Liu et al. constructed a ceRNA network associated to AF susceptibility and persistence, and then prioritized the selected lncRNAs using an innovative application of the improved RWR-M algorithm. Myocardial infarction-associated transcripts (MIAT) and LINC00964 were identified as featured lncRNAs in the network. These discoveries are in line with a prior study (Deshmukh et al., 2015), and they also suggested that ceRNA network analysis might shed light on the basic mechanisms underlying AF and offer promising therapeutic and diagnostic tools. In another study, kidney stones are hypothesized to be caused by Randall’s plaque (RP) (Daudon et al., 2015). Xia et al. investigated the rate of immune cell infiltration as well as the ceRNA network in RPs from renal stone patients and validated the results both *in vivo* and *in vitro*. They then found that the lncRNA-associated differentially expressed mRNAs were significantly associated with renal interstitial fibrosis in extracellular matrix tissues, regulatory cell responses to growth factor stimulation, and collagen-containing extracellular matrices. In addition, Wu et al. summarized the recent research progress of LINC00467, detailing its biological mechanisms as an oncogene and clinical values as a prognostic predictor in various types of cancers.

MiRNAs as a class of small ncRNAs play a crucial role in downregulating gene expression by competitively interacting with the mRNA 3′UTR region and mediating RNA degradation through the RISC complex (Zhou et al., 2022). MiRNAs have two main applications. On the one hand, artificial miRNAs are often developed as genetic tools based on their downregulation mechanism to affect gene expression (Zhu et al., 2018). On the other hand, miRNAs are often utilized in targeted gene therapy due to the characteristic that miRNA targets can be used to attain tissue or cell-type specific transgene expression (Luo et al., 2015a).

MiRNAs have important roles in the emergence of epilepsy due to their capacity to regulate a variety of mRNAs. Previous studies have shown that miRNAs could alter neuronal excitability, which has an impact on the incidence of epilepsy (Henshall et al., 2016). Su et al. constructed a miRNA-mRNA regulation network that regulates ion channel genes in the mesial temporal lobe epilepsy (mTLE). They identified that miR-27a-3p might control a number of ion channel genes associated with mTLE, pointing to the possibility of using it as a diagnostic biomarker for mTLE.

In addition, the human induced pluripotent stem cells (hiPSCs) and embryonic stem cells (hESCs) are powerful tools for genetic studies in the recent years (Luo et al., 2021a). Some previous studies have illustrated the roles of several lncRNA-miRNA-mRNA circuits in regulating the pluripotent genes in hESCs (Wang et al., 2013; Xu et al., 2016). In this Research Topic, Chen et al. systematically reviewed the current progress in applying iPSC-derived models to study the regulatory role of miRNAs in developmental processes.

As sequencing technologies advance quickly, the expression profiles of different classes of ncRNAs are feasible to be characterized simultaneously in disease models, such as dilated cardiomyopathy (DCM) (Lin et al., 2021). Based on sex differences in DCM, Liu et al. constructed an immune-related ceRNA network including 5 lncRNAs, 6 miRNAs, and 29 mRNAs that might regulate several immune-related signaling pathways. Among these genes, CBL, CXCL12, and IL6ST were found to be connected to immune cell infiltration.

Here we assembled a compendium of 11 manuscripts including nine original researches and two reviews that cover the scope of this Research Topic, summarizing the critical aspects of the gene regulation mediated by competing RNAs in various diseases.

Additionally, we would also like to highlight other novel forms of competing RNA-mediated gene regulation that are yet to be included in the above compendium. Guide RNA (gRNA) is an important class of artificial small ncRNA that competitively bind with elements on the genome with the CRISPR/Cas complex (Luo et al., 2015b). Besides genome editing, the RNA-guided gene targeting technology has also been exploited quickly and extensively for diverse purposes, including gene regulation (Luo et al., 2016).

Recently, RNA viruses, such as SARS-CoV-2, have been found to act as exogenous competing RNAs and thus regulate endogenous gene expression, which may contribute to the disease progression (Bertolazzi et al., 2020). And in the current COVID-19 pandemic, it is of great interest to focus on the role of SARS-CoV-2 in competing RNA-mediated gene regulation (Luo et al., 2021b).

## References

[B1] Bertolazzi G. Cipollina C. Benos P. V. Tumminello M. Coronnello C. (2020). miR-1207-5p can contribute to dysregulation of inflammatory response in COVID-19 via targeting SARS-CoV-2 RNA. Front. Cell. Infect. Microbiol. 10, 586592. 10.3389/fcimb.2020.586592 33194826 PMC7658538

[B2] Daudon M. Bazin D. Letavernier E. (2015). Randall’s plaque as the origin of calcium oxalate kidney stones. Urolithiasis 43 (S1), 5–11. 10.1007/s00240-014-0703-y 25098906

[B3] Deshmukh A. Barnard J. Sun H. Newton D. Castel L. Pettersson G. (2015). Left atrial transcriptional changes associated with atrial fibrillation susceptibility and persistence. Circ. Arrhythm. Electrophysiol. 8 (1), 32–41. 10.1161/CIRCEP.114.001632 25523945 PMC4334691

[B4] Fontana G. See E. Pandit A. (2015). Current trends in biologics delivery to restore intervertebral disc anabolism. Adv. Drug Deliv. Rev. 84, 146–158. 10.1016/j.addr.2014.08.008 25174310

[B5] Henshall D. C. Hamer H. M. Pasterkamp R. J. Goldstein D. B. Kjems J. Prehn J. H. M. (2016). MicroRNAs in epilepsy: pathophysiology and clinical utility. Lancet. Neurol. 15 (13), 1368–1376. 10.1016/S1474-4422(16)30246-0 27839653

[B6] Hindricks G. Potpara T. Dagres N. Arbelo E. Bax J. J. Blomström-Lundqvist C. (2021). 2020 ESC Guidelines for the diagnosis and management of atrial fibrillation developed in collaboration with the European Association for Cardio-Thoracic Surgery (EACTS): The Task Force for the diagnosis and management of atrial fibrillation of the European Society of Cardiology (ESC) Developed with the special contribution of the European Heart Rhythm Association (EHRA) of the ESC. Eur. Heart J. 42 (5), 373–498. 10.1093/eurheartj/ehaa612 32860505

[B7] Lin Z. Zhao Y. Dai F. Su E. Li F. Yan Y. (2021). Analysis of changes in circular RNA expression and construction of ceRNA networks in human dilated cardiomyopathy. J. Cell. Mol. Med. 25 (5), 2572–2583. 10.1111/jcmm.16251 33484110 PMC7933965

[B8] Luo Y. Zhu D. (2014). Combinatorial control of transgene expression by hypoxia-responsive promoter and MicroRNA regulation for neural stem cell-based cancer therapy. Biomed. Res. Int. 2014, 751397. 10.1155/2014/751397 24864258 PMC4016878

[B9] Luo Y. Zhu D. Lam D. H. Huang J. Tang Y. Luo X. (2015a). A double-switch cell fusion-inducible transgene expression system for neural stem cell-based antiglioma gene therapy. Stem Cells Int. 2015, 649080. 10.1155/2015/649080 26074975 PMC4436471

[B10] Luo Y. Zhu D. Zhang Z. Chen Y. Sun X. (2015b). Integrative analysis of CRISPR/Cas9 target sites in the human *HBB* gene. Biomed. Res. Int. 2015, 514709. 10.1155/2015/514709 25918715 PMC4396065

[B11] Luo Y. Xu X. An X. Sun X. Wang S. Zhu D. (2016). Targeted inhibition of the miR-199a/214 cluster by CRISPR interference augments the tumor tropism of human induced pluripotent stem cell-derived neural stem cells under hypoxic condition. Stem Cells Int. 2016, 3598542. 10.1155/2016/3598542 27965712 PMC5124688

[B12] Luo Y. Huang J. Tang Y. Luo X. Ge L. Sheng X. (2019). Regional methylome profiling reveals dynamic epigenetic heterogeneity and convergent hypomethylation of stem cell quiescence-associated genes in breast cancer following neoadjuvant chemotherapy. Cell Biosci. 9 (1), 16. 10.1186/s13578-019-0278-y 30774927 PMC6367786

[B13] Luo Y. Chen Y. Zhang M. Ma X. Zhu D. Chen Y. (2021a). Generation of an induced pluripotent stem cell line GZHMCi008-A derived from a patient with SRY-positive 46, XX testicular disorder of sex development. Stem Cell Res. 57, 102583. 10.1016/j.scr.2021.102583 34710837

[B14] Luo Y. Zhang M. Chen Y. Chen Y. Zhu D. (2021b). Application of human induced pluripotent stem cell-derived cellular and organoid models for COVID-19 research. Front. Cell Dev. Biol. 9, 720099. 10.3389/fcell.2021.720099 34552930 PMC8450444

[B15] Oichi T. Taniguchi Y. Oshima Y. Tanaka S. Saito T. (2020). Pathomechanism of intervertebral disc degeneration. JOR Spine 3 (1), 1076. 10.1002/jsp2.1076 PMC7084053 32211588

[B16] Panni S. Lovering R. C. Porras P. Orchard S. (2020). Non-coding RNA regulatory networks. Biochim. Biophys. Acta. Gene Regul. Mech. 1863 (6), 194417. 10.1016/j.bbagrm.2019.194417 31493559

[B17] Peng F. Gong W. Li S. Yin B. Zhao C. Liu W. (2021). circRNA_010383 acts as a sponge for miR-135a, and its downregulated expression contributes to renal fibrosis in diabetic nephropathy. Diabetes 70 (2), 603–615. 10.2337/db20-0203 33472945

[B18] Salmena L. Poliseno L. Tay Y. Kats L. Pandolfi P. P. (2011). A ceRNA hypothesis: the rosetta stone of a hidden RNA language? Cell 146 (3), 353–358. 10.1016/j.cell.2011.07.014 21802130 PMC3235919

[B19] Tang S. Tan G. Jiang X. Han P. Zhai B. Dong X. (2016). An artificial lncRNA targeting multiple miRNAs overcomes sorafenib resistance in hepatocellular carcinoma cells. Oncotarget 7 (45), 73257–73269. 10.18632/oncotarget.12304 27689326 PMC5341977

[B20] Tay Y. Kats L. Salmena L. Weiss D. Tan S. M. Ala U. (2011). Coding-independent regulation of the tumor suppressor PTEN by competing endogenous mRNAs. Cell 147 (2), 344–357. 10.1016/j.cell.2011.09.029 22000013 PMC3235920

[B21] Tay Y. Rinn J. Pandolfi P. P. (2014). The multilayered complexity of ceRNA crosstalk and competition. Nature 505 (7483), 344–352. 10.1038/nature12986 24429633 PMC4113481

[B22] Visci G. Tolomeo D. Agostini A. Traversa D. Macchia G. Storlazzi C. T. (2020). CircRNAs and Fusion-circRNAs in cancer: New players in an old game. Cell. Signal. 75, 109747. 10.1016/j.cellsig.2020.109747 32860952

[B23] Wang Y. Xu Z. Jiang J. Xu C. Kang J. Xiao L. (2013). Endogenous miRNA sponge lincRNA-RoR regulates Oct4, Nanog, and Sox2 in human embryonic stem cell self-renewal. Dev. Cell 25 (1), 69–80. 10.1016/j.devcel.2013.03.002 23541921

[B24] Xu C. Zhang Y. Wang Q. Xu Z. Jiang J. Gao Y. (2016). Long non-coding RNA GAS5 controls human embryonic stem cell self-renewal by maintaining NODAL signalling. Nat. Commun. 7, 13287. 10.1038/ncomms13287 27811843 PMC5097163

[B25] Zhang Z. K. Li J. Guan D. Liang C. Zhuo Z. Liu J. (2018). A newly identified lncRNA MAR1 acts as a miR-487b sponge to promote skeletal muscle differentiation and regeneration: MAR1 sponges miR-487b to promote muscle differentiation. J. Cachexia Sarcopenia Muscle 9 (3), 613–626. 10.1002/jcsm.12281 29512357 PMC5989759

[B26] Zhang Y. Yang M. Yang S. Hong F. (2022). Role of noncoding RNAs and untranslated regions in cancer: A review. Medicine 101 (33), e30045. 10.1097/MD.0000000000030045 35984196 PMC9388041

[B27] Zhou G. Zhang M. Zhang J. Feng Y. Xie Z. Liu S. (2022). The gene regulatory role of non-coding RNAs in non-obstructive azoospermia. Front. Endocrinol. 13, 959487. 10.3389/fendo.2022.959487 PMC9436424 36060931

[B28] Zhu D. Zhao Z. Cui G. Chang S. Hu L. See Y. X. (2018). Single-cell transcriptome analysis reveals estrogen signaling coordinately augments one-carbon, polyamine, and purine synthesis in breast cancer. Cell Rep. 25 (8), 2285–2298. 10.1016/j.celrep.2018.10.093 30463022

